# Disseminated *Mycobacterium chimaera* Following Open-Heart Surgery, the Heater–Cooler Unit Worldwide Outbreak: Case Report and Minireview

**DOI:** 10.3389/fmed.2020.00243

**Published:** 2020-06-16

**Authors:** Emmanuel Lecorche, Gauthier Pean de Ponfilly, Faiza Mougari, Hanaa Benmansour, Elodie Poisnel, Frederic Janvier, Emmanuelle Cambau

**Affiliations:** ^1^Université de Paris, IAME, INSERM, UMR1137, UFR de Médecine, Paris, France; ^2^CNR-MyRMA, Centre National de Reference pour les Mycobactéries et les Antituberculeux, APHP, Paris, France; ^3^APHP, Hôpital Lariboisière, Service de Microbiologie, Paris, France; ^4^Service de Medecine Interne, Hôpital d'Instruction des Armées Sainte Anne, Toulon, France; ^5^Service de microbiologie, Hôpital d'Instruction des Armées Sainte Anne, Toulon, France; ^6^Ecole du Val-de-Grâce, Paris, France

**Keywords:** HCU, cardiac surgery, non-tuberculous mycobacteria, NTM, spondylodiscitis

## Abstract

Invasive cardiovascular infections by *Mycobacterium chimaera* associated with open-heart surgery have been reported worldwide since 2013. Here, we report a case of a 61 year old man, without any other particular medical background, who underwent cardiac surgery for replacing part of the ascending aorta by a bio-prosthetic graft. Eighteen months later, the patient was painful at the lower back with fever. A pyogenic vertebral osteomyelitis due to *M. chimaera* associated to graft infection was diagnosed after 6 months of sub-acute infection. The patient presented a disseminated disease with cerebral lesions, chorioretinitis, and chronic renal failure. Despite adequate antimicrobial treatment and graft explantation, the patient died after 6 years. We reviewed the literature on *M. chimaera* infections associated with open-heart surgery. The worldwide outbreak has been explained by airborne bioaerosol generated by the 3T heater–cooler unit (HCU) used during cardiac by-pass surgical procedures. These infections are difficult to diagnose because of a long latency period (up to several years), with no specific symptoms and a highly specialized microbiological diagnosis. The treatment is based on antibiotics and surgery. These infections are also difficult to treat, since the mortality rate is high around 50%. Prevention is necessary by modifying the use of HCUs in operating rooms.

## Background

Invasive cardiovascular infections due to *M. chimaera* secondary to open-heart surgery were first described in 2015 in Switzerland (1). These infections are difficult to diagnose because of non-specific symptoms, a difficult microbiological diagnostic, and a poor prognostic. They were attributed to contamination from the heater-cooler units (HCU) present in the operating rooms since similar strains of *M. chimaera* were found in their water tanks. In addition, since the strains from several patients, who underwent surgery at different periods, were also similar, a common reservoir was sought. Since 2015, cases were reported worldwide not only in Europe (Switzerland, Germany, Netherlands, England, France, Italia, Spain, and Ireland) (1–6), but also in North America (United-States, Canada) (7, 8), Hong-Kong (9), New-Zeeland, and Australia (10). Most of the cases were due to the same epidemic strain similar to those found also in the HCUs (11). The epidemic strain has also been found in HCUs in China (12). In 2015, the European Center for Disease Prevention and Control issued a Rapid Risk Assessment (13) and the Food and Drug Administration published as well a safety communication about infections associated with heater-cooler devices and recommendations to deal with the risk (14).

Here, we report the case of a patient diagnosed in France for a *M. chimaera* infection following cardiac surgery, and subsequently reviewed the literature about this outbreak and discussed the patient case.

## Case Presentation

A 61 year old man, without any particular medical background, underwent cardiac surgery in 2012 for replacing part of the ascending aorta by a bio-prosthetic graft and repairing the aortic arch due to a type I aortic dissection. During immediate follow up, a local infection was diagnosed at the coronary angiography insertion site. Since it was show to be caused by *Proteus mirabilis* and *Pseudomonas aeruginosa*, the patient was treated with 10 days of ceftazidime.

**Figure 1:**
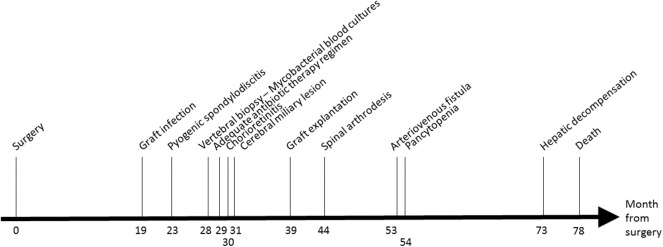
Timeline of the patient's clinical course after cardiac surgery.

Eighteen months after the surgery (M18, see Figure 1), the patient presented fewer with a lower back pain that was intensified for 1 month. A positron emission tomography/computed tomography (PET-CT) suspected a graft infection with a pseudoaneurysm para-aortic. A transthoracic echocardiography did not show any signs of endocarditis and blood cultures remained negative. An empirical treatment was initiated with piperacillin-tazobactam, teicoplanin, and rifampicin.

**Figure 2:**
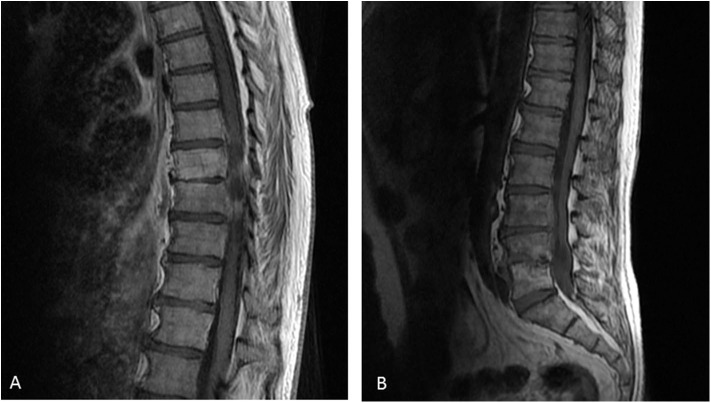
Vertebral magnetic resonance imaging of the vertebral lesions. Vertebral magnetic resonance imaging revealed lesions of the vertebral bodies at T8-T9-L4-L5-S1 and invertebral disks between T8-T9 **(A)** and L4-L5-S1 **(B)**, with an epidural abscess of 5 cm at the L3 and L4 levels, consistent with a pyogenic vertebral osteomyelitis.

At M24, vertebral magnetic resonance imaging (MRI) revealed lesions of the vertebral bodies at T8-T9-L4-L5-S1 and intervertebral disks between T8-T9 and L4-L5-S1, with an epidural abscess of 5 cm at the L3 and L4 levels, consistent with a pyogenic vertebral osteomyelitis (Figure 2). That was consistent with the PET-CT results showing metabolic activity around the peri-aortic graft in favor of infection. Transcutaneous vertebral biopsies, made at M27, were culture-positive for acid fast bacilli (AFB) after 21 days incubation on 7H9 liquid medium (BACT/ALERT® MP, Biomerieux) and subsequently on Lowenstein Jensen solid medium (Bio-Rad). The AFB isolate was identified first as *M. intracellulare* by GenoType® Mycobacterium CM (Hain Lifescience), and subsequently confirmed as *M. chimaera* by GenoType® NTM-DR (Hain Lifescience), ITS and *hsp65* sequencing. Susceptibility testing of the isolate was performed using a commercial microdilution method, SLOMYCO Myco Sensititre™ (Thermo Scientific™) and showed a wild type susceptibility pattern with a minimal inhibitory concentration (MIC) of clarithromycin at 2 mg/L, MIC of amikacin at 8 mg/L, MIC of linezolid at 32 mg/L and MIC of moxifloxacin at 4 mg/L. Three mycobacterial blood cultures performed at the same period were also positive for *M. chimaera*. The patient was treated with a 4-antibiotic regimen combining azithromycin, ethambutol, rifampicin, and moxifloxacin. The risk associated with graft explantation was felt to be prohibitively high, and the decision was therefore made to proceed with conservative management.

At M 29, 2 months after antimicrobial therapy has started, an ocular examination showed a bilateral chorioretinitis associated with uveitis of the left eye. At M30, a MRI of the brain, performed because of confusion, revealed diffuse hypersignals of both hemispheres consistent with cerebral miliary lesions. The patient showed then a worsening of the vertebral lesions and of the renal function. Because of the disseminated infection, it was decided to replace the aortic graft at M39. *M. chimaera* was isolated from explanted prosthetic tissues.

At M44, the patient underwent arthrodesis of thoracic spine. Due to the chronic renal failure, an arteriovenous fistula was created at M53. The patient showed pancytopenia at M72. At M73, the patient suffered from hepatic and neurologic decompensation. Unfortunately, the patient died at M78.

## The Worldwide Outbreak of Disseminated *M. Chimaera* Infection Associated to Open-Heart Surgery

### The Causative Agent

*M. chimaera* is a slow growing non-tuberculous mycobacterium (NTM) belonging to the *Mycobacterium avium* complex (MAC) (15) and was first described in 2004 (16). Like *M. avium* and *M. intracellulare, M. chimaera* is predominantly seen in immunocompromised patients and in pulmonary infections in patients with chronic lung diseases. Among patients with sputum culture-positive with MAC, the patients with *M. chimaera* were less likely to meet criteria for infection than *M. avium* and *M. intracellulare*, suggesting a lower virulence or a different reservoir (17, 18). The natural reservoir of *M. chimaera* is not well-known and is supposed to be similar to other species of MAC. MAC can be found in distribution water systems (19) and *M. chimaera* was found frequently in household water (20). Drug susceptibility patterns of *M. chimaera* are comparable to those of the MAC with modal MIC of 2 mg/L for clarithromycin, 0.5 mg/L for rifabutin, 4–8 mg/L for rifampicin and ethambutol, 8 mg/L for amikacin, 4 mg/L for moxifloxacine, and 32 mg/L for linezolid (21).

### Burden and Impact of the Disease

Over 120 cases of post-cardiac surgery *M. chimaera* infections have been reported worldwide. More cases are to be expected since many countries did not register any cases. Using data from Switzerland, the incidence of *M. chimaera* disseminated disease associated with open heart surgery was estimated to 156–282 cases per year in the 10 major cardiac valve replacement market countries (22). Using data from the national British investigation, the risk of *M. chimaera* infection for person who underwent cardiothoracic surgery significantly increased since 2012. Out of 10,000 patients undergoing open heart surgery, 300–400 were estimated to experience endocarditis by 5 years post-surgery and one to develop *M. chimaera* infection (2). A long latency period (median of 21 months) was observed between cardiac surgery and symptoms (23). The reported mortality rate for these infections was remarkably high, around 50% (24, 25). No cases were reported in healthcare workers related to the HCU epidemic. However, one case of *M. chimaera* pulmonary infection has been described in a healthcare worker previously exposed to HCU (26). Other devices such as thermoregulatory devices used for extracorporeal membrane oxygenation (ECMO) may be also at risk of transmission, but no cases were reported (27).

### Transmission Routes

**Figure 3:**
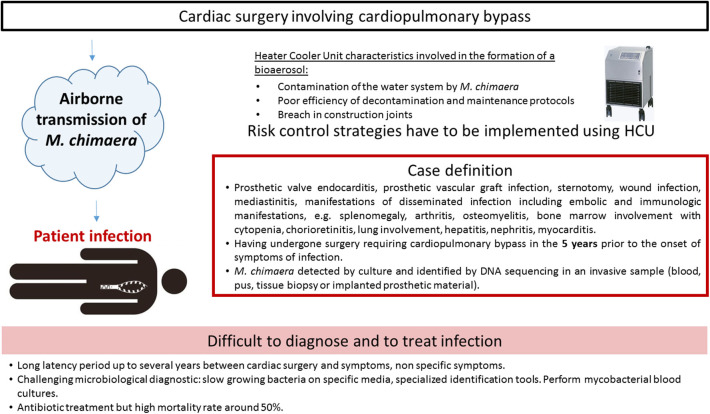
*Mycobacterium chimaera* infections associated with open heart surgery characteristics.

The first report described two cases, caused by closely related *M. chimaera* strains, as assessed by randomly amplified polymorphic DNA (RAPD)-PCR. The two patients had heart surgery 2 years apart from each other (28). Due to identical RAPD-PCR patterns, a deeper investigation was retrospectively conducted and six cases were finally detected in the institution (1). The prospective on-site observations and microbiological sampling in the hospital environment showed that *M. chimaera* was present in water circuits of the LivaNova HCU (25). HCU are essential components of cardiopulmonary bypass operation used during open-chest heart surgery. They are connected to the extracorporeal circuit enabling the warming of the patient's blood and the cooling of the cardioplegia solution, since tanks are filled with water. Water in the circuits does not come into direct contact with the patient. Interestingly, air sampling cultures in the operating room were also positive with *M. chimaera* when a HCU was running, but not when it was turned off. Laser particle measurement and microbial air cultures confirmed that during operation, mycobacterial particles were dispersed from the contaminated HCU into the air of the operating room via aerosolization, despite ultraclean air ventilation (27). The aerosol was generated through a breach in construction joints on the tank cover and released into the operating environment via the rear cooling fan, thereby causing infection (10). Phylogenetic analysis by whole genome sequencing (WGS) showed a strong clustering of all *M. chimaera* epidemic isolates. They were indeed clonal isolates around the world in Europe, North America, or Australia (2, 29). Moreover, this cluster comprised isolates from patients, HCUs and water at the HCU industry production site (30). The most plausible hypothesis of such genetic similarity is that the devices were contaminated by a point source when manufactured before being sent to the cardiac surgery wards. Contamination and infections characteristics are presented in Figure 3.

### Risk Factors

In the majority of cases, patients had undergone cardiac valve or aortic vascular graft surgery prior to diagnosis. However, patients who have undergone other operations that involve cardiopulmonary bypass, including heart or lung transplantation and introduction of ventricular assist devices are also at risk (23). To our knowledge, no cases have been described with other devices than LivaNova, formerly Sorin. It has been shown that the odds of NTM infection increase with the duration time a patient is exposed to a running HCU. The risk reached statistical significance for surgery time longer than 5 h (29). Survival analysis measured for a cohort of 30 cases identified several factors associated with better survival: younger age, mitral valve surgery, mechanical valve replacement, higher serum sodium concentration (30).

## Diagnosis and Treatment

### Clinical Manifestations

Patients exhibited a wide spectrum of disease including surgical site infection, e.g., prosthetic valve endocarditis (PVE), aortic graft infection or localized thoracic infection, as well as disseminated infection with diverse presentations, such as bacteraemia, osteomyelitis or other bone lesions, cholestatic hepatitis, granulomatous nephritis. Disseminated disease with encephalitis have also been described (31). Patients most commonly complained of non-specific symptoms such as fever, malaise, weight loss, cough, or dyspnea. Laboratory findings included cytopenia and elevated inflammatory markers, transaminase and creatinine blood levels (24). Eye involvement was correlated with the course of the systemic disease. Patients with few choroidal lesions had a favorable outcome, whereas all patients with widespread chorioretinitis died of systemic complications (32). Complications of *M. chimaera* infection are listed in Table 1.

**Table 1:** Complications of *M. chimaera* infection.

**Constitutional symptoms**	Fever, fatigue, weight loss, night sweats, joint pain, shortness of breath
**Cardiac**	Prosthetic valve endocarditis and/or prosthetic vascular graft infection, pseudoaneurysm, aortic root abscess, aortic dissection, myocarditis
**Localized infections**	Surgical site infection, sternotomy wound infection mediastinitis
**Embolic and immunologic manifestations**	Splenomegaly, hepatitis, nephritis, disseminated granulomatous disease
**Ocular infection**	Chorioretinitis, panuveitis
**Bone infection and bone marrow involvement**	Cytopenia, osteomyelitis, spondylodiscitis arthritis

### Diagnosis

The European Center for Disease Prevention (33) and the American Center for Disease Control and Prevention (34) have formulated a case definition for *M. chimaera* infections associated with open heart surgery based on three criteria: (i) any of the clinical criteria, including prosthetic valve or vascular infection, localized infection, and disseminated infection, (ii) an exposure criteria, e.g., having undergone surgery requiring cardiopulmonary bypass in the 5 years prior to the onset of symptoms of infection, (iii) microbiological criteria, e.g., *M. chimaera* detected by culture or identified by DNA sequencing in an invasive sample. A 5 years period from surgery to presentation of symptoms has been mentioned, but the delay before diagnosis can be longer, the longest reported time being more than 6 years (35).

Mycobacterial cultures remain the essential investigation for all sample types: blood, tissue and bone biopsy, pus, and urine. Culture of *M. chimaera* from peripheral blood is the most common method of microbiological diagnosis (30). Its sensitivity increases by performing multiple samples: 3 sets of mycobacterial blood cultures on different days are recommended by the English guidance (36). It is essential to inform the laboratory of the possibility of *M. chimaera* infection, in order to ensure samples are taken into the correct containers, such as blood culture bottle specific for mycobacterial growth (e.g., BACTEC™ Myco/F Lytic). Molecular technologies based on acid nucleic amplification can be used, especially on sterile samples (tissue biopsies, vascular graft) positive for acid fast bacilli at microscopic examination.

*M. chimaera* identification is challenging. It is slow growing in liquid and solid media, so growth detection may take between 2 and 8 weeks. Most laboratories can identify a *M. chimaera* isolate as a species of the *M. avium* complex (MAC), but precise speciation needs specialized tests (15), such as PCR sequencing of the internal transcribed spacer (ITS) sequence between 16S and 23S ribosomal DNA. *M. chimaera* is closely related to *M. intracellulare*, for instance they show only a single nucleotide difference in 16S ribosomal DNA sequences. *M. chimaera* can be misidentified as *M. intracellulare* by mass spectrometry (MALDI-TOF MS) or some commercial DNA hybridization probe assays (37). In fact, *M. chimaera* has been extensively classified as *M. intracellulare* before 2004 (18). Analysis of WGS is necessary to assess whether a clinical strain is related to the HCU outbreak strain (11). Microbiologic results should be considered alongside histopathology findings, e.g., detection of non-caseating granuloma and foamy macrophages with acid fast bacilli in cardiac or vascular tissues, prosthetic material, or in specimen from the sternotomy wound.

Transesophageal echocardiography (TOE) was shown to be more sensitive than transthoracic echocardiography in diagnosing *M. chimaera* PVE (3). TOE can be normal at presentation, while the patient later went on be diagnosed with PVE (30). Normal echocardiogram alone cannot be used to exclude infection and serial assessment should be considered. Computed tomography (CT) can assess aortic graft infection. Moreover PET-CT provides additional evidence in which standard CT has been equivocal and has also proved useful in the diagnosis of *M. chimaera* spondylodiscitis and PVE.

### Differential Diagnosis

The presentation and laboratory features of the disease can be very similar to sarcoidosis (29). *M. chimaera* investigations should be undertaken in all patients for whom a diagnosis of sarcoidosis is being considered and who has an appropriate history of cardiothoracic surgery.

### Treatment

The optimal treatment for *M. chimaera* HCU-related infections is not known. The combination of clarithromycin or azithromycin, rifampin, or rifabutin and ethambutol is the treatment regimen designed on the basis of that recommended for MAC lung disease (38). Antimicrobial susceptibility of *M. chimaera* strains isolated during the outbreak were fortunately all susceptible to clarithromycin and amikacin (3, 39). Given the disseminated nature of HCU-related infections and the poor outcome, parenteral amikacin, and fluoroquinolones were added (24, 40). According to guidelines, a minimum of 12 months therapy is indicated for non-HIV patients with disseminated MAC disease (38). The optimal duration of therapy in HCU-related infections is unknown and some patients required treatment for more than 24 months. Blood drug concentrations have to be monitored, a study reporting that half of the patients did not reached optimal drug levels (3). The removal of prosthetic materials was associated with a lower risk of mortality for classical PVE (41). Given technical difficulties and the risk of surgery, such decision has to be discussed with the cardiovascular surgeon in case of invasive infection with *M. chimaera* (30).

### Preventive Measures

Several measures were proposed to minimize the risks of *M. chimaera* infections. The manufacturer updated its disinfection recommendations in 2015 with more frequent cleaning and disinfections of the water system (42). In addition, the manufacturer proposed complete refurbishment and replacement of the internal tubing of devices. Despite intensified cleaning and disinfection, surveillance samples from factory-new units still grew *M. chimaera* (43). Environmental testing and microbiological screening of HCUs could be performed. However, there is no standardization with regard to the collection of samples and the laboratory methods used (44).

Due to the difficulties to maintain water with good microbiological quality, different strategies were implemented in hospitals (45). The most definitive option was to remove HCUs from the operating room, though this may not be feasible for all facilities. A custom-made airtight housing for the HCU was also used in order to contain the bioaerosol. If a definitive mitigation strategy cannot be implemented, HCU should be oriented so that the aerosol from the exhaust is directed in the opposite way from the patient. However, the utility of this strategy is unproven and may continue to place the patient at risk (12).

## Discussion

*M. chimaera* was initially described in respiratory samples (16). Only one case of vertebral osteomyelitis was reported in a patient with prednisolone treatment, without history of cardiac surgery (46). Cases of disseminated *M. chimaera* infections were rare until 2015 (25). The involvement of this species in disseminated disease was quite unusual and led to the description of this outbreak associated with cardiac surgery. In the case of our patient, the surgery was performed in 2012 when the risk due to contaminated HCU was not known yet. The microbial diagnosis was rapidly done when the spondylodiscitis diagnosis was made, and isolation and identification of *M. chimaera* was done in a laboratory with expertise. The strain was genomically sequenced and was shown to cluster with the epidemic isolates. No sample was obtained from the HCU in this hospital at the time of the contamination and later. Although an adequate antimicrobial treatment was immediately given, the patient died 78 months later. We may think that an earlier replacement of aortic graft could have helped in the cure of the infection, but surgery could have been also lethal (40).

This case was the only one registered in this area of France, a second case being registered in the Paris area in 2010 were detailed elsewhere (47).

## Data Availability Statement

The data sequencing are now available on the NCBI platform (PRJNA576780, SAMN13008644, SRR10256732).

## Ethics Statement

Written informed consent was obtained from the individual's next of kin for the publication of any potentially identifiable images or data included in this article.

## Author Contributions

EL, GP, and EC contributed to the manuscript. GP review the clinical case and EL reviewed the literature. EP took care of the patient. FM, HB, and FJ provide clinical and microbiological data. EC supervised this work.

## Conflict of Interest

The authors declare that the research was conducted in the absence of any commercial or financial relationships that could be construed as a potential conflict of interest.
